# A palliative care approach in psychiatry: clinical implications

**DOI:** 10.1186/s12910-020-00472-8

**Published:** 2020-04-19

**Authors:** Mattias Strand, Manne Sjöstrand, Anna Lindblad

**Affiliations:** 1 Stockholm Centre for Eating Disorders, Wollmar Yxkullsgatan 27B, 118 50 Stockholm, Sweden; 2 Centre for Psychiatry Research, Department of Clinical Neuroscience, Karolinska Institutet, & Stockholm Health Care Services, Stockholm County Council, 171 77 Stockholm, Sweden; 3 Stockholm Centre for Healthcare Ethics, Department of Learning, Informatics, Management and Ethics, Karolinska Institutet, 171 77 Stockholm, Sweden

**Keywords:** Palliative care, Psychiatry, Schizophrenia, Anorexia nervosa, Borderline personality disorder, Recovery

## Abstract

**Background:**

Traditionally, palliative care has focused on patients suffering from life-threatening somatic diseases such as cancer or progressive neurological disorders. In contrast, despite the often chronic, severely disabling, and potentially life-threatening nature of psychiatric disorders, there are neither palliative care units nor clinical guidelines on palliative measures for patients in psychiatry.

**Main text:**

This paper contributes to the growing literature on a palliative approach in psychiatry and is based on the assumption that a change of perspective from a curative to a palliative approach could help promote patient-centeredness and increase quality of life for severely ill patients in psychiatry as well as in somatic medicine. To exemplify this, we offer three different clinical scenarios: severe and enduring anorexia nervosa, treatment-refractory schizophrenia, and chronic suicidality and persistent self-injury in borderline personality disorder.

**Conclusion:**

We emphasize that many typical interventions for treatment-refractory psychiatric disorders may indeed be of a palliative nature. Furthermore, introducing traditional features of palliative care, e.g. so-called goals of care conversations, could aid even further in ensuring that caregivers, patients, and families agree on which treatment goals are to be prioritized in order to optimize quality of life in spite of severe, persistent mental disorder.

## Background

In psychiatry as well as in other medical disciplines, clinicians sometimes encounter situations in which curative treatment is not possible. For instance, patients suffering from severe and enduring psychiatric disorders, such as major affective or psychotic disorders, may develop a chronic and treatment-refractory condition where recovery in a traditional, curative sense is essentially out of reach. Similar situations may arise in patients suffering from a combination of severe somatic and psychiatric disorders or patients who have developed secondary psychiatric conditions due to somatic disease. Factors such as old age and physical or cognitive impairment may further complicate the situation, not least by interfering with the feasibility of providing pharmacological or electroconvulsive treatment [1–3].

In recent years, there has been a growing academic interest in palliative approaches in severe and persistent mental illness. More specifically, the discussion has focused on whether psychiatric care could benefit from explicitly acknowledging that a curative treatment is not always possible in major psychiatric disorders, and whether a focus on symptom reduction and quality of life may sometimes be a better option, even in potentially life-threatening conditions [1–16]. According to these ideas, clinical psychiatry could learn from palliative care in somatic medicine by adopting a similar approach to patients suffering from psychiatric conditions beyond cure. The suggested approach is often labelled ‘palliative psychiatry’ and a working definition of the concept, based on the World Health Organization (WHO) definition of palliative care [17], has been presented by Trachsel and colleagues in 2016 and in subsequent papers [3, 11, 13].

According to the WHO, palliative care “is an approach that improves the quality of life of patients and their families facing the problem associated with life-threatening illness, through the prevention and relief of suffering by means of early identification and impeccable assessment and treatment of pain and other problems, physical, psychosocial and spiritual” [17]. This perspective is as relevant to psychiatry as it is to somatic medicine, and concerns patients with severe persistent psychiatric disorders as well as the often multifactorial conditions encountered within geriatric psychiatry [3, 11, 13]. Analogous to palliation in somatic medicine, a palliative care approach in psychiatry does not mean merely giving up the attempts of finding a curative treatment [3]. Furthermore, in order for it to be a meaningful concept, it needs to entail an active approach with specific and attainable treatment goals [3]. However, palliative care in psychiatry is a comparatively novel concept and thus, except for case studies and a survey of clinicians’ attitudes, empirical research on its clinical applicability is still lacking [3, 4, 10, 12]. Moreover, it is yet to be spelled out what specifically distinguishes palliative care from other care options in psychiatry [3, 13].

In accordance with previous research, we believe that a change of perspective from a curative to a palliative approach could help promote patient-centeredness and increase quality of life for patients with severe and enduring psychiatric disorders [3, 18]. However, as we have argued elsewhere, we do not believe that there is a need for a separate definition of palliative care in psychiatry [13]. Hence, we base our discussion here on the definition of palliative care established by the WHO [13, 17].

In the following, we first explore how a palliative approach could be applied in three different non-exhaustive clinical scenarios: severe and enduring anorexia nervosa, treatment-refractory schizophrenia, and chronic suicidality and persistent self-injury in borderline personality disorder. Second, we discuss some specific questions related to the concept of palliative care in psychiatry, including staging of mental illness, the role of so-called goals of care conversations, and the overlap between palliative care and other care concepts in psychiatry.

### Severe and enduring anorexia nervosa

Various suggestions for how to operationalize severe and enduring anorexia nervosa (in terms of chronicity, severity, treatment resistance, etc.) exist in the literature and it can therefore be difficult to estimate how prevalent the condition is. It is often stated that roughly 20–25% of patients with anorexia nervosa do not experience remission on long-term follow-up [19, 20]. In the past decades, a large number of papers have addressed the issue of medical futility in the treatment of patients with severe anorexia nervosa [4–8, 12, 21]. These papers have, however, mainly focused on the most extreme clinical scenarios where death from undernutrition is imminent and where terminating treatment usually results in the patient subsequently dying from ensuing cardiac arrest or multiple organ dysfunction. A much more common clinical dilemma in the treatment of severe and enduring anorexia nervosa is how to deal with the patient who has ‘tried everything’ without success; i.e., cases of long-standing illness and a more or less permanent low body-mass index (BMI) where numerous treatment episodes have not resulted in any lasting remission [22]. If these patients are seen as suffering from a lack of motivation for treatment and recovery, they may be dismissed from treatment and left to fend for themselves, which often results in subsequent deterioration and a renewed need for intensified treatment. A vicious cycle may ensue, where repeated acute treatment episodes merely lead to partial remission, whereupon the patient is again dismissed because of insufficient treatment motivation—a scenario that may add to the overall picture of futility and frustration. Alternatively, a fear of ending treatment may lead to continued interventions that neither patient, nor therapist, actually believes in, for lack of better options.

One way to resolve this stalemate could be to change the treatment goals, so that the focus is explicitly on symptom control and quality of life, rather than on actual remission. The current case management model for patients with a severe and enduring eating disorder at the Stockholm Centre for Eating Disorders may serve as an illustration [23]. Here, patients with a more than 10-year history of disordered eating and numerous previous unsuccessful treatment attempts are on a case-by-case basis assigned a case manager who coordinates further interventions, such as somatic controls and laboratory check-ups, vocational rehabilitation, family support, etc. Should the patient temporarily need more intensive somatic or psychiatric treatment, the case manager helps in planning the intervention and maintains contact with the patient and other health care providers. Importantly, the treatment focus is not on normalized eating or weight restoration—indeed, many of the patients in this program continuously maintain a low BMI. Instead, the explicit aim is to help patients achieve a higher quality of life and a lower risk of medical complications *in spite of* their mental illness.

The decision to make the transition from traditional curative treatment to the case management program is preceded by a rigorous assessment procedure in close collaboration with the individual patient, whereby patient preferences and needs are explored and family and social networks are charted. After this in-depth assessment, the formal decision to change treatment perspectives is finally explicitly presented to the patient in what could be seen as a form of goals of care conversation, although that specific term is not used.

Similar models of care, based on assertive community treatment, for patients with long-standing anorexia nervosa who have opted out of traditional treatment have been described from Vancouver [24] and Toronto [16]. Here too, “the success of [the program] is not measured by how much weight an individual has gained or whether they cease binge eating and purging, but rather on an improved quality of life while still having an eating disorder and an ability to be maintained in the community” ([16], p. 225).

If, at first glance, programs such as these do not appear to constitute a specifically palliative approach, it should be remembered that anorexia nervosa has one of the highest mortality rates among psychiatric disorders. In a large meta-analysis [25], the standardized mortality ratio in anorexia nervosa was 5.2, which is significantly higher than rates reported in schizophrenia, bipolar disorder, or unipolar depression. Not least, a markedly increased risk for suicide has been found in patients with anorexia nervosa [25, 26]. Treatment-refractory anorexia nervosa is certainly a life-threatening condition. The clinical models outlined above, with clear focus on quality of life rather than cure or even symptom reduction, not only support patients in the process towards a satisfying life despite a severe disease but also meet the criteria of palliative care as stated in the WHO definition [17].

Even so, the decision to switch from a curative to a palliative approach need not be irreversible. Interestingly, our clinical experience from the Stockholm Centre for Eating Disorders model described above is that the focus on increased quality of life has in some cases resulted in revived prospects for curative treatment. It appears that when patients with long-lasting mental illness achieve a higher level of functioning in everyday life as a result of switching to a palliative route, some may also experience a reignited motivation to engage in traditional cure-focused interventions.

### Treatment-refractory schizophrenia

Despite being a low prevalence disorder, schizophrenia is noted as the 12th leading cause of disability on a global scale [27]. Nowadays, much effort is put into preventing the transition from first-episode psychosis into schizophrenia [28]. Moreover, it has been suggested that as much as half of schizophrenia patients recover or significantly improve in the long run [29]. Even so, schizophrenia often takes a chronic, life-long course and many patients live with the disorder into old age [30]. In schizophrenia, a final stage characterized by multiple relapses and severe, persistent, or unremitting illness with worsening impact on life has been described, with recommendations for a pharmacological emphasis on clozapine, the use of other tertiary treatments, and a focus on social participation despite ongoing disability [31]. Clozapine has comparably high response rates in treatment refractory schizophrenia; nevertheless, it is not a catch-all solution and due to the risk of bothersome and potentially lethal adverse reactions a change of medication is suggested if no effect is seen after a 6-month treatment trial [32]. Unfortunately, experimental high-dose, combination, or off-label medication regimens are not uncommon in treatment-refractory patients [2], resulting in polypharmacy and an increased risk of adverse effects such as insulin resistance and metabolic syndrome [33].

Furthermore, each failed treatment trial risks augmenting a sense of despair and hopelessness in patients and family members [2]. Instead, explicitly switching to a palliative treatment route—with continued pharmacological maintenance treatment in reasonable doses in combination with an increased focus on symptom management and quality of life interventions—could potentially instil hope, increase autonomy, and improve overall outcomes for patients with treatment-refractory schizophrenia or other chronically disabling psychotic disorders [3].

Interventions such as cognitive-behavior therapy, social skills training, or the recently developed AVATAR therapy [34] aimed at patients with longstanding psychotic disorders fit well within this framework, as their focus is usually on symptom management (such as tackling negative symptoms and coping with paranoid delusions) and social inclusion (such as reducing social avoidance and reviving the interest for daily activities) [35]. For example, acceptance and commitment therapy has been shown to reduce the need for rehospitalization as well as the belief in the validity of hallucinations in patients with psychotic disorders [36]. Goals such as these may prove more useful in treatment-refractory schizophrenia than continued attempts at reducing positive symptoms [2].

### Chronic suicidality and persistent self-injury in borderline personality disorder

Borderline personality disorder (BPD) usually does not follow a trajectory of progressive deterioration [2]. In fact, even though acute behavioral symptoms may come and go, several prospective studies have shown that most individuals with BPD experience remission over time and that many recover fully over the course of their lives [37]. Still, a minority of patients will present with continuous treatment-refractory symptoms of chronic suicidality and persistent self-injurious behaviors with few signs of improvement over time [38].

Recurrent thoughts of suicide and suicidal behaviors are a diagnostic feature of BPD [39], tend to be unrelated to any comorbid affective disorder, and do not typically improve substantially with antidepressants [38]. Evidence suggests that those who make repeated suicide attempts and those who actually die from suicide are in fact two distinct groups, albeit with a significant overlap; for example, suicide completers are older, more likely to be male, and more often suffer from a non-affective psychotic disorder [40]. There is also evidence to suggest that for those who engage in repetitive self-injurious behaviors, a reduction in the effort needed to engage in self-harm occurs over time (e.g., by pain habituation and personal identification with self-harm as a coping strategy) so that the behaviors risk becoming self-sustaining [41].

Moreover, a conceptual distinction can be made between suicidal and non-suicidal self-injury (the latter often abbreviated as NSSI). In patients engaging in NSSI, self-injury may, for example, serve a purpose of affect regulation, self-punishment, anti-dissociation, or interpersonal communication of agony and despair [42]. However, it is important to acknowledge that suicide attempts are also common in those individuals who engage in NSSI [43] and that identifying the precise underlying function of self-injury is not always a straightforward task.

The helpfulness of repeated hospitalization of patients with BPD at times of suicidal communication or urges to self-harm has often been questioned [44, 45]. Usually, it is argued that in a context of chronic suicidality—in contrast to, for example, suicidality in an individual with a severe depressive episode—suicide is actually not prevented by hospitalization and that this may instead result in negative effects, such as regressive behaviors and ‘psychiatrization’ [44]. In Sweden, there has been a debate about the practice of referring patients with chronic suicidality and severe persistent self-injury to high-security compulsory care units, whereby a vicious cycle of escalating self-injurious behaviors and increasingly intrusive preventive interventions may ensue [46].

In contrast, for certain patients with treatment-refractory symptoms of chronic suicidality and persistent severe self-injurious behaviors, a palliative approach to psychiatric care and suicide prevention could possibly result in a de-escalation of self-harm and an increased quality of life. Interventions of this type could include educating patients in first aid skills and basic anatomy in order to avoid permanent bodily damage or sepsis [47], or promoting safer methods of self-injury (e.g., avoiding ligatures or overdoses) [48]. In part, this builds on the finding that even though self-injury is associated with an increased risk of suicide on group level, for the individual patient it may actually serve as a coping mechanism that protects against overwhelming urges to attempt suicide [42, 49]. The overall aim is still to reduce the impact of self-injury on patients’ lives [50], while pointing to the fact that insisting on a ‘zero tolerance’ policy regarding NSSI could potentially put patients in greater risk of an actual suicide attempt [47, 51]. Of course, it should also be recognized that for a patient engaging in NSSI, learning about anatomy might be helpful in avoiding serious injury, whereas for a patient with actual suicidal intent, knowledge of anatomy could hypothetically increase the risk of lethal self-injury. These considerations notwithstanding, the idea of ‘assisting’ patients in their self-injurious behaviors is most certainly in direct conflict with many practitioners’ fundamental beliefs about what psychiatry ought to strive towards [48]. Such objections should not be taken lightly; however, one might also want to keep in mind that most interventions aimed at harm reduction have initially met with large scepticism [52]. (For further arguments for and against a harm reduction paradigm in self-injury, see also [53, 54].)

It should, however, be mentioned that negative stereotyping and stigmatization of individuals with BPD may also result in overzealous categorical withholding of inpatient treatment [55]. Notably, brief episodes of self-admission to inpatient treatment have been used as an intervention aimed at harm reduction and increased quality of life for patients with BPD and self-injurious behaviors [56, 57].

Of our three example scenarios, this is probably the most controversial. In suggesting a palliative approach to the treatment of patients with BPD and continuous treatment-refractory symptoms of chronic suicidality and persistent self-injurious behaviors, we are not implying that there is no possibility of further cure. What we do suggest is that certain situations point to a potential need of change in perspectives, where an explicitly palliative approach—including, for example, such interventions as offering quick access to brief inpatient admission in times of need, adjusting medication focusing on symptom management, promoting less dangerous ways of coping with suicidal or self-injurious urges (including safer means of actual self-injury), and finding ways to increase quality of life in spite of continuous suicidal ideation—could be helpful in de-escalating a vicious circle of treatment failures, despair, and trying to achieve a brief respite from hopelessness through self-injury. In parallel to the description of a palliative approach in the treatment of severe and enduring anorexia nervosa above, such a shift in perspective could in fact eventually lead to a more stable situation where renewed efforts towards psychotherapeutic treatment may be more realistic, although this is not an explicit aim of the palliative paradigm.

### From curative to palliative treatment goals—when and how?

The scenarios above are not exhaustive in any sense, but nevertheless raise several questions associated with the application of a palliative care approach in psychiatry. In the following, we address some of these, focusing mainly on staging, the role of goals of care conversations, and the overlap between palliative care and other care concepts in psychiatry.

In somatic medicine, palliative care is routinely initiated in cases of life-threatening disease when it is not (or no longer) possible to modify it by intervention. It should be noted, however, that incurability is not a sufficient condition; some chronic diseases, such as type 1 diabetes mellitus, may be life-threatening if untreated, but perfectly compatible with a long and otherwise healthy life as long as proper treatment is provided. In such scenarios, although a definitive cure for the underlying disorder is not possible, treatment still aims at prolonging life. Such objectives are clearly distinct from the treatment goals as defined in palliative care, where the aim is typically to reduce symptoms and increase quality of life while neither hastening nor postponing death [17].

When assessing illness severity in somatic medicine, physicians may use staging guidelines based on understanding of the disease trajectory in specific, well-defined diseases [58]. Through staging, severity in terms of risk for death or impairment can be assessed and clinical decision-making concerning treatment options supported [58, 59]. However, when it comes to psychiatric disorders, no generally accepted guidelines for staging exist [1–3, 15, 59]. Several staging models have been developed [31, 59–61], but critical voices have been raised [15]. Not least, the course of mental illnesses varies substantially; some conditions are episodic and others continuously progressive, etc.

This heterogeneity is important for the question of which treatment goals are realistic. Since psychiatric disorders appear on a spectrum, different individuals with the same diagnosis may present with different clinical manifestations in terms of symptom intensity, duration, and recurrence [2]. Moreover, patients respond differently to treatment. Therefore, assessing the longitudinal severity of mental illness—i.e., the prognosis—may often be less straightforward than in other medical disciplines. Undoubtedly, this makes the transition from curative to palliative care goals difficult, and poses a major challenge for the introduction of a palliative approach in psychiatry. As others before us, we see a need for further research and for the development of reliable staging guidelines in psychiatry [2, 3, 60].

As stated before, palliative care in psychiatry in the form presented in the scenarios above does not represent an entirely new approach for treatment. Its general features are derived from a patient-centered approach, based on informed consent and shared decision making, which is relevant for all healthcare [3, 17]. As a comparison, patients in somatic medicine are generally informed—explicitly and in-depth in a so-called *goals of care conversation*—when the focus of treatment is switched from a curative to a palliative approach [62–64] (in Swedish, this is usually referred to as a ‘breakpoint conversation’, accentuating the overall changes in perspectives regarding the prospects of cure). We believe that this basic principle should also be applied when care goals are switched from curative to palliative in psychiatry. However, as described above, there are currently no formal recommendations or guidelines on how to decide when a psychiatric condition is to be regarded as treatment-refractory, or how this clinical watershed should be communicated to the patient. Importantly, in emphasizing the importance of explicit goals of care conversations, it is also fully possible to imagine *reverse* goals of care conversations where it is decided to abandon the palliative approach in favor of renewed attempts at cure; e.g., in the case of altered patient circumstances or the introduction of new treatment options.

We realize that the mere use of the term ‘palliative’ may be considered controversial [2, 10]. In fact, in one of the programs described above, this terminology is to be explicitly avoided (“[case management] is not to be seen as a ‘last resort’ or as palliative care” ([21] , p. 4), presumably because many patients will instinctively think of palliative treatment as an intervention that connotes an impending death. These connotations may also affect clinicians, not least in psychiatric practice where much effort is usually made towards minimizing the risk of death. For example, it has been shown that many geriatric psychiatrists prefer “being ‘seen to treat’, even when success was doubtful, because this provided the most defensible approach to practice” ([30], p. 586). Thus, engaging patients in goals of care conversations or other interventions described in palliative terms may not necessarily be seen as an intrinsic part of a psychiatrist’s work duties. The use of any specific terminology involving explicit references to palliation is certainly not the vital part of the argument; indeed, we realize that the mere act of labeling an approach as palliative could initially worry patients and families as well as health care professionals—although a recent survey among Swiss psychiatrists suggests that the concept may in fact not be all that controversial [10]. Even so, acknowledging the importance of even daring to apply a palliative perspective in the treatment of patients with severe mental illness could in many cases lead to more favorable outcomes [3, 11].

### Palliative care, harm reduction, and recovery

Due to the broad heterogeneity in the outcome of psychiatric disorders, complete recovery, in terms of absence of symptoms and return to premorbid functioning, is not always a realistic goal [65, 66]. A distinction between recovery *from* and recovery *in* a disorder has been suggested. Whereas recovery from a disorder is synonymous with a traditional notion of cure—i.e., complete remission—the concept of recovery in a disorder, often referred to as the recovery model, implies that even though the patient still fulfils diagnostic criteria for a certain psychiatric disorder, s/he has access to necessary tools in order to be able to manage symptoms of mental illness and lead a fulfilling life in spite of not being formally cured [65]. Davidson and Roe write: “Unlike in most physical illnesses, people may consider themselves to be ‘in’ this form of recovery while continuing to have, and be affected by, mental illness.” ([63] , p. 462) Analogous to this view of recovery, Trachsel and colleagues emphasize two models, *clinical* and *personal* recovery, in their discussion of palliative care in psychiatry [3]. Although the recovery model is not specifically described in terms of palliation, it provides an accurate outline of potential aims of palliative care in psychiatry for many individuals with treatment-refractory psychiatric disorders [3]. The recovery model originally has its roots in the consumer-advocacy movement, emphasizing lived experience of recovery and hope [66]. Although a unifying definition of the concept is lacking, it is often described in terms of “a process that involves gaining or regaining many aspects of life that are usually taken for granted and may have been lost or severely compromised by mental illness” ([67], p. 40).

With its focus on self-determination and quality of life in spite of enduring mental illness, the recovery model shares common grounds with palliative care. According to Trachsel and colleagues, the recovery model targets a similar group of patients as in the palliative approach, and palliative care in psychiatry should be “understood as functioning in conjunction with other approaches oriented towards prevention, curation, rehabilitation, or recovery” ([3], p. 4). Furthermore, it is suggested that a palliative approach may support a patient’s recovery in a disorder [3]. We readily agree; however, we would like to underscore that while the recovery model may apply to *any* patient with a disabling mental disorder, palliative care should be reserved specifically for those suffering from life-threatening conditions for which recovery *from* as well as recovery *in* the disorder may remain distant despite high-quality psychiatric treatment [65]. Whereas the recovery model offers an overall change in perspective on what it means to live with mental illness (or, for that matter, chronic somatic illness), palliative care should be seen as a highly specialized approach to medical and nursing care with a robust framework in terms of medical interventions, patient involvement, and ethics. We believe that applying a palliative approach in psychiatry would make it possible to go beyond the valuable insights offered by the recovery model and focus specifically on establishing ‘hands-on’ tools and guidelines comparable to those in somatic palliative care.

In this context, it is also worth distinguishing palliative care in psychiatry from the concept of harm reduction [10], i.e., interventions aimed at reducing negative consequences of problematic behaviors such as substance use without necessarily fully extinguishing the underlying behaviors themselves [68]. Harm reduction techniques can certainly be applied as part of a palliative approach to mental health, as seen in the clinical scenarios described above. However, the scope of palliative care in psychiatry is much broader [10] and does not need to involve any harm reduction interventions at all if they are not required in order to meet the goals outlined in the WHO definition of palliative care [17]. Obviously, the notions of palliation, recovery, and harm reduction may at times overlap and we hope for further discussions about the interrelated nature of these models.

## Conclusion

We have illustrated how a palliative approach in psychiatry could be implemented in three different clinical scenarios: severe and enduring anorexia nervosa, treatment-refractory schizophrenia, and chronic suicidality and severe persistent self-injury in borderline personality disorder. The type of interventions referred to as palliative are by no means ‘novel’ and ‘cutting-edge’—quite the contrary, we interpret palliative care as an approach defined by its goals and not by the use of specific treatments.

Furthermore, we argue that the introduction of goals of care conversations in psychiatry could aid even further in ensuring that patients and families understand the approach taken and feel safe in knowing what treatment goals are helpful and attainable.

Finally, we discuss the overlap between palliative care, harm reduction and recovery, and conclude that in contrast to the other approaches, palliative care should be reserved specifically for those suffering from severe, persistent life-threatening conditions.

## Data Availability

Not applicable.

## Abbreviations

BMI: Body-mass index
BPD: Borderline personality disorder
NSSI: Non-suicidal self-injury
WHO: World Health Organization

## References

[1] Berk M, Singh A, Kapczinski F (2008). When illness does not get better: do we need a palliative psychiatry?. Acta Neuropsychiatr.

[2] Berk M, Berk L, Udina M, Moylan S, Stafford L, Hallam K (2012). Palliative models of care for later stages of mental disorder: maximizing recovery, maintaining hope, and building morale. Aust New Zeal J Psychiatry..

[3] Trachsel M, Irwin SA, Biller-Andorno N, Hoff P, Riese F (2016). Palliative psychiatry for severe persistent mental illness as a new approach to psychiatry? Definition, scope, benefits, and risks. BMC Psychiatry.

[4] Lopez A, Yager J, Feinstein RE (2010). Medical futility and psychiatry: palliative care and hospice care as a last resort in the treatment of refractory anorexia nervosa. Int J Eat Disord..

[5] O’Neill J, Crowther T, Sampson G (1994). Anorexia nervosa: palliative care of terminal psychiatric disease. Am J Hosp Palliat Med.

[6] Russon L, Alison D (1998). Does palliative care have a role in treatment of anorexia nervosa? Palliative care does not mean giving up. BMJ..

[7] Westmoreland P, Mehler PS (2016). Caring for patients with severe and enduring eating disorders (SEED): certification, harm reduction, palliative care, and the question of futility. J Psychiatr Pract.

[8] Williams CJ, Pieri L, Sims A (1998). Does palliative care have a role in treatment of anorexia nervosa? We should strive to keep patients alive. BMJ..

[9] Yager J (2015). The futility of arguing about medical futility in anorexia nervosa: the question is how would you handle highly specific circumstances?. Am J Bioeth.

[10] Trachsel M, Hodel MA, Irwin SA, Hoff P, Biller-Andorno N, Riese F (2019). Acceptability of palliative care approaches for patients with severe and persistent mental illness: a survey of psychiatrists in Switzerland. BMC Psychiatry..

[11] Trachsel M, Irwin SA, Biller-Andorno N, Hoff P, Riese F (2016). Palliative psychiatry for severe and persistent mental illness. Lancet Psychiatry.

[12] Trachsel M, Wild V, Biller-Andorno N, Krones T (2015). Compulsory treatment in chronic anorexia nervosa by all means? Searching for a middle ground between a curative and a palliative approach. Am J Bioeth.

[13] Lindblad A, Helgesson G, Sjöstrand M (2019). Towards a palliative care approach in psychiatry: do we need a new definition?. J Med Ethics.

[14] McGrath P, Holewa H. Mental health and palliative care: exploring the ideological interface. Int J Psychosoc Rehabil. 2004;9:107–19.

[15] Trauer T (2012). Palliative models of care for later stages of mental disorder: Maximising recovery, maintaining hope and building morale. Aust New Zeal J Psychiatry..

[16] Kaplan AS, Miles A, Touyz S, Le Grange D, Lacey JH, Hay P (2016). The role of palliative Care in Severe and Enduring Anorexia Nervosa. Managing severe and enduring anorexia nervosa: a Clinician’s guide.

[17] World Health Organization (2019). WHO Definition of Palliative Care.

[18] Swiss Academy of Medical Sciences (SAMS). Medical-ethical guidelines and recommendations on palliative care. Basel: Swiss Academy of Medical Sciences; 2013.

[19] Ciao AC, Accurso EC, Wonderlich SA, Touyz S, Le Grange D, Lacey JH, Hay P (2016). What do we know about severe and enduring anorexia nervosa?. Managing severe and enduring anorexia nervosa: a Clinician’s guide.

[20] Dobrescu SR, Dinkler L, Gillberg C, Råstam M, Gillberg C, Wentz E (2020). Anorexia nervosa: 30-year outcome. Br J Psychiatry.

[21] Geppert CMA (2015). Futility in chronic anorexia nervosa: a concept whose time has not yet come. Am J Bioeth.

[22] Wildes JE, Forbush KT, Hagan KE, Marcus MD, Attia E, Gianini LM, et al. Characterizing severe and enduring anorexia nervosa: an empirical approach. Int J Eat Disord. 2017;50:389–97. 10.1002/eat.22651 PMC5386793 27991694

[23] Molin M, von Hausswolff-Juhlin Y, Norring C, Hagberg L, Gustafsson SA (2016). Case management at an outpatient unit for severe and enduring eating disorder patients at Stockholm Centre for Eating Disorders-- a study protocol. J Eat Disord.

[24] Williams KD, Dobney T, Geller J (2010). Setting the eating disorder aside: an alternative model of care. Eur Eat Disord Rev.

[25] Keshaviah A, Edkins K, Hastings ER, Krishna M, Franko DL, Herzog DB (2014). Re-examining premature mortality in anorexia nervosa: a meta-analysis redux. Compr Psychiatry.

[26] Keel PK, Dorer DJ, Eddy KT, Franko D, Charatan DL, Herzog DB (2003). Predictors of mortality in eating disorders. Arch Gen Psychiatry.

[27] Diminic S, Ferrari AJ, Santomauro DF, Whiteford HA, Charlson FJ, Scott JG (2018). Global epidemiology and burden of schizophrenia: findings from the global burden of disease study 2016. Schizophr Bull.

[28] Millan MJ, Andrieux A, Bartzokis G, Cadenhead K, Dazzan P, Fusar-Poli P (2016). Altering the course of schizophrenia: progress and perspectives. Nat Rev Drug Discov.

[29] Vita A, Barlati S (2018). Recovery from schizophrenia: is it possible?. Curr Opin Psychiatry.

[30] Relyea E, MacDonald B, Cattaruzza C, Marshall D (2019). On the margins of death: a scoping review on palliative care and schizophrenia. J Palliat Care.

[31] McGorry PD, Hickie IB, Yung AR, Pantelis C, Jackson HJ (2006). Clinical staging of psychiatric disorders: a heuristic framework for choosing earlier, safer and more effective interventions. Aust New Zeal J Psychiatry..

[32] Siskind D, McCartney L, Goldschlager R, Kisely S (2016). Clozapine v. first- and second-generation antipsychotics in treatment-refractory schizophrenia: systematic review and meta-analysis. Br J Psychiatry.

[33] Correll CU, Frederickson AM, Kane JM, Manu P (2007). Does antipsychotic polypharmacy increase the risk for metabolic syndrome?. Schizophr Res.

[34] Craig TKJ, Rus-Calafell M, Ward T, Leff JP, Huckvale M, Howarth E (2018). AVATAR therapy for auditory verbal hallucinations in people with psychosis: a single-blind, randomised controlled trial. Lancet Psychiatry.

[35] Leff J, Warner R (2006). Social inclusion of people with mental illness.

[36] Bach P, Gaudiano BA, Hayes SC, Herbert JD (2013). Acceptance and commitment therapy for psychosis: intent to treat, hospitalization outcome and mediation by believability. Psychosis..

[37] Temes CM, Zanarini MC (2018). The longitudinal course of borderline personality disorder. Psychiatr Clin North Am.

[38] Paris J (2004). Half in love with easeful death: the meaning of chronic Suicidality in borderline personality disorder. Harv Rev Psychiatry.

[39] American Psychiatric Association (2013). DSM-5: diagnostic and statistical manual of mental illness.

[40] Beautrais AL (2001). Suicides and serious suicide attempts: two populations or one?. Psychol Med.

[41] Liu RT (2017). Characterizing the course of non-suicidal self-injury: a cognitive neuroscience perspective. Neurosci Biobehav Rev.

[42] Klonsky ED (2007). The functions of deliberate self-injury: a review of the evidence. Clin Psychol Rev.

[43] Victor SE, Klonsky ED (2014). Correlates of suicide attempts among self-injurers: a meta-analysis. Clin Psychol Rev.

[44] Paris J (2004). Is hospitalization useful for suicidal patients with borderline personality disorder?. J Personal Disord.

[45] National Institute for Health and Clinical Excellence. Borderline Personality Disorder: Treatment and Management. NICE clinical guideline 78. London: National Institute for Health and Clinical Excellence; 2009. 39480982

[46] Åkerman S, Eriksson T (2012). Slutstation rättspsyk: Om tvångsvårdade kvinnor som inte dömts för brott.

[47] Sullivan PJ (2017). Should healthcare professionals sometimes allow harm? The case of self-injury. J Med Ethics.

[48] James K, Samuels I, Moran P, Stewart D (2017). Harm reduction as a strategy for supporting people who self-harm on mental health wards: the views and experiences of practitioners. J Affect Disord.

[49] Lindgren B-M, Öster I, Åström S, Graneheim UH (2011). ‘They don’t understand … you cut yourself in order to live’. Interpretative repertoires jointly constructing interactions between adult women who self-harm and professional caregivers. Int J Qual Stud Health Well-being.

[50] Gutridge K (2010). Safer self-injury or assisted self-harm?. Theor Med Bioeth.

[51] Edwards SD, Hewitt J (2011). Can supervising self-harm be part of ethical nursing practice?. Nurs Ethics.

[52] Des Jarlais DC (2017). Harm reduction in the USA: the research perspective and an archive to David purchase. Harm Reduct J.

[53] Sullivan PJ (2018). Sometimes, not always, not never: a response to Pickard and Pearce. J Med Ethics.

[54] Pickard H, Pearce S (2017). Balancing costs and benefits: a clinical perspective does not support a harm minimisation approach for self-injury outside of community settings. J Med Ethics.

[55] Kealy D, Ogrodniczuk JS (2010). Marginalization of borderline personality disorder. J Psychiatr Pract.

[56] Strand M, von Hausswolff-Juhlin Y (2015). Patient-controlled hospital admission in psychiatry: a systematic review. Nord J Psychiatry.

[57] Helleman M, Goossens PJJ, Kaasenbrood A, van Achterberg T (2014). Experiences of patients with borderline personality disorder with the brief admission intervention: a phenomenological study. Int J Ment Health Nurs.

[58] Gonnella JS, Hornbrook MC, Louis DZ (1984). Staging of disease: a case-mix measurement. JAMA..

[59] Cosci F, Fava GA (2013). Staging of mental disorders: systematic review. Psychother Psychosom.

[60] Ruhé HG, van Rooijen G, Spijker J, Peeters FPML, Schene AH (2012). Staging methods for treatment resistant depression. A systematic review. J Affect Disord.

[61] Treasure J, Stein D, Maguire S (2015). Has the time come for a staging model to map the course of eating disorders from high risk to severe enduring illness? An examination of the evidence. Early Interv Psychiatry.

[62] The Swedish Register of Palliative Care. Vad bör ett brytpunktssamtal innehålla och när ska det genomföras? 2015. http://media.palliativ.se/2015/08/Brytpunkt20111.pdf. Accessed 28 Mar 2019.

[63] The Swedish National Board of Health and Welfare. Nationellt kunskapsstöd för god palliativ vård i livets slutskede. Stockholm, SWE; 2013. https://www.socialstyrelsen.se/globalassets/sharepoint-dokument/artikelkatalog/kunskapsstod/2013-6-4.pdf. Accessed 28 Mar 2019.

[64] Bernacki RE, Block SD (2014). Force for the AC of PHVCT. Communication about serious illness care goals: a review and synthesis of best practices. JAMA Intern Med.

[65] Davidson L, Roe D (2007). Recovery from versus recovery in serious mental illness: one strategy for lessening confusion plaguing recovery. J Ment Health.

[66] Dawson L, Rhodes P, Touyz S (2014). The recovery model and anorexia nervosa. Aust New Zeal J Psychiatry.

[67] McKellar D, Ng F, Chur-Hansen A (2016). Is death our business? Philosophical conflicts over the end-of-life in old age psychiatry. Aging Ment Health.

[68] Hawk M, Coulter RWS, Egan JE, Fisk S, Reuel Friedman M, Tula M (2017). Harm reduction principles for healthcare settings. Harm Reduct J.

